# Let-7g* and miR-98 Reduce Stroke-Induced Production of Proinflammatory Cytokines in Mouse Brain

**DOI:** 10.3389/fcell.2020.00632

**Published:** 2020-07-17

**Authors:** David L. Bernstein, Slava Rom

**Affiliations:** ^1^Department of Pathology and Laboratory Medicine, Lewis Katz School of Medicine, Temple University, Philadelphia, PA, United States; ^2^Center for Substance Abuse Research, Lewis Katz School of Medicine, Temple University, Philadelphia, PA, United States

**Keywords:** microRNA, let-7, cytokines, leukocyte-brain infiltration, blood brain barrier

## Abstract

Stroke is a debilitating illness facing healthcare today, affecting over 800,000 people and causing over 140,000 deaths each year in the United States. Despite being the third-leading cause of death, very few treatments currently exist for stroke. Often, during an ischemic attack, the blood-brain barrier (BBB) is significantly damaged, which can lead to altered interactions with the immune system, and greatly worsen the damage from a stroke. The impaired, BBB promotes the infiltration of peripheral inflammatory cells into the brain, secreting deleterious mediators (cytokines/chemokines) and resulting in permanent barrier injury. let-7 microRNAs (miRs) are critical for regulating immune responses within the BBB, particularly after ischemic stroke. We have previously shown how transient stroke decreases expression of multiple let-7 miRs, and that restoration of expression confers significant neuroprotection, reduction in brain infiltration by neutrophils, monocytes and T cells. However, the specific mechanisms of action of let-7 miRs remain unexplored, though emerging evidence implicates a range of impacts on cytokines. In the current study, we evaluate the impacts of miR-98 and let-7g* on targeting of cytokine mRNAs, cytokine release following ischemic stroke, and cell-specific changes to the neurovascular space. We determined that miR-98 specifically targets IP-10, while let-7g* specifically aims IL-8, and attenuates their levels. Both produce strong impacts on CCL2 and CCL5. Further, let-7g* strongly improves neurovascular perfusion following ischemic stroke. Together, the results of the study indicate that let-7 miRs are critical for mediating endothelial-immune reactions and improving recovery following ischemic stroke.

## Introduction

Stroke exerts a tremendous burden in lives, financial costs, and healthcare. Over 800,000 people have strokes each year in the United States and over 140,000 of them will die as a result, representing roughly one death out of every 20. Stroke is also a very expensive disease; the cost per hospitalization was ∼$20,000 in 2003–06 (Wang et al., 2014) and the total financial burden is estimated at 219 billion dollars each year (Yang et al., 2017). Despite such prevalence, very few treatments currently exist for stroke, and the ones that are available (namely tPA and mechanical thrombectomy), contain significant side effects and contraindications. Further, neither offers protection from reperfusion injury, which arises from inflammation originating in the damaged blood vessels where the stroke occurred (Nour et al., 2013; Ahnstedt et al., 2016). This is a critical issue; inflammatory disease processes are a central element of the pathophysiology of stroke (Lambertsen et al., 2012). Inflammatory machinery is very important in the pathophysiologic processes following the onset of ischemic stroke, and cytokines are key players in the inflammatory mechanism and contribute to ischemia-reperfusion damage progression. As such, vascular-immune interactions represent a critical avenue for potential future stroke therapeutics. The let-7 family of microRNAs (miRs) is integral for vascular function, and considered strongly neuroprotective (Sen et al., 2009; Jolana and Kamil, 2017). let-7 miRs are critical for mediating the cellular response to inflammation. Low or impaired expression is associated with greater cellular damage (Rom et al., 2015; Li et al., 2019), while increased expression of let-7 miRs is associated with improved cellular function (Zhang et al., 2016), and resistance to oxidative stress (Hou et al., 2012). Within this family, two miRs appear to be particularly important for the vascular response to ischemic stroke: miR-98 and let-7g*. These are critical mediators of brain endothelial cells and are strongly downregulated during inflammatory events (Rom et al., 2015).

let-7 miRs may be particularly important in the progression of stroke. Following ischemic or hemorrhagic stroke, cytokine expression is significantly altered (Lambertsen et al., 2012), often lasting for many days (Tarkowski et al., 1997; Nayak et al., 2012). These changes to cytokine levels can profoundly alter the interactions between the endothelial cells comprising the vasculature and activated leukocytes and lymphocytes of the immune system (Pawluk et al., 2020), leading to changes in barrier permeability, and worsening the size of the stroke penumbra. Cytokines such as CCL2, CCL3, CCL5, and CXCL-1 have been particularly implicated in such processes (Dimitrijevic et al., 2006; Yang et al., 2019). let-7 miRs may be critical for regulating such processes, as they can strongly alter the expression of stroke-impacted cytokines (Rom et al., 2015; Jickling et al., 2016).

Restoration of endogenous expression of miR-98 (Bernstein et al., 2019) or let-7g*(Bernstein et al., 2020) can prevent a significant degree of damage following ischemia. However, the mechanisms through which this occurs may be widely different. Although both miRs can reduce the size of the ischemic penumbra, as well as the number of immune cells which extravasate into the infarcted region (Bernstein et al., 2019, 2020), there are important differences. miR-98 is associated with the integrity of tight junction proteins such as ZO-1 and claudin-5 (Zhuang et al., 2016), and protection against Cas-3-dependant apoptotic pathways (Li et al., 2015), while let-7g* modulates interactions with low density lipoproteins (Liu et al., 2017), and impedes apoptosis through Akt-dependent mechanisms (Joshi et al., 2016). The differences in gene targets is particularly important with regards to inflammation. miR-98 is associated with expression of IL-10 (Liu et al., 2011; Li et al., 2018, 2019), while let-7g is associated with the response to IGF-B signaling (Zhou et al., 2015; Huang et al., 2017), and with IL-6 (Huang et al., 2017). Such differences are critical for mediating the type of immune cells recruited following stroke, as well as the nature of their interactions with the endothelial cells of the BBB. However, such interactions do not fully explain the nature of the vascular changes.

In our previous investigations into the role of let-7 miRs in regulating post-stroke recovery, we determined that miR-98 overexpression reduces the infiltration of monocytes into the ischemic brain, and appears to attenuate the activation of microglia into a proinflammatory state (Bernstein et al., 2019). Conversely, let-7g* was more effective at limiting the infiltration of neutrophils, and other forms of T-cells (Bernstein et al., 2020). These results, combined with the diversity of other mRNA targets of the two let-7 miRs, indicate that let-7 miRs are capable of conferring neuroprotection from stroke through a wide range of cellular mechanisms. In addition, as let-7 miRs can produce pro- and anti-inflammatory effects, it is critical to better understand the specific actions of its constituent members, in order to guide future therapeutics for stroke. For this report, we characterized the specific mechanisms through which let-7g* and miR-98 promote recovery from ischemic stroke. We correlate sequence binding with the differing nature of cytokine release, as well as with structural changes to the neurovasculature. Both miRs are important for maintaining BBB integrity, and managing inflammation. With this study, we investigate the specific pathways through which this occurs.

## Materials and Methods

### Animals

All animal experiments were approved by the Temple University Institutional Animal Care and Use Committee and were conducted in accordance with Temple University guidelines, which are based on the National Institutes of Health (NIH) guide for care and use of laboratory animals and in the ARRIVE (Animal Research: Reporting *in vivo* Experiments) guidelines (study design, experimental procedures, housing and husbandry, and statistical methods)^1^. 10-week old male C57BL/6 mice were purchased from the Jackson Laboratory (Bar Harbor, ME, United States) and given *ad libitum* access to food and water. Animals were kept in a 12 h light/12 h dark cycle for the duration of experiments. Animals were group-housed prior to surgery, and single-housed thereafter.

### Transient Middle Cerebral Artery Occlusion (tMCAO) and miR Delivery

Mice were subjected to 60 min focal cerebral ischemia produced by transient intraluminal occlusion with a monofilament made of 6–0 nylon with a rounded tip (Doccol Corp., Sharon, MA, United States, cat# 602312PK10) into the middle cerebral artery (MCAO) as described previously (Jin et al., 2010a, b; Engel et al., 2011). Sham-operated mice were subjected to the same surgical procedure, but the filament was not advanced far enough to occlude the MCA. We then used a protocol recently developed in our laboratory (Rom et al., 2015; Bernstein et al., 2019) to mix 5 nmol synthetic miRNA with Lipofectamine 2000 (Life Technologies, Carlsbad, CA, United States) in RNase and DNase-free water (Life Technologies) prior to retroorbital injection in 100 uL sterile PBS (Bernstein et al., 2019, 2020).

### Enzyme-Linked Immunosorbent Assay (ELISA)

Animals were sacrificed via intracardiac perfusion at 72 h following tMCAO, and brain hemispheres were extracted and dissolved in 400 uL lysis buffer (RayBiotech, Norcross, GA, United States). Following centrifuging at 10,000 × *g* for 10 min, supernatant was collected, and analyzed for cytokine level via multi-plex ELISA, in accordance with standard methods (Weng and Zhao, 2015). A sample titration curve was performed prior to all assays in order to determine the optimal dilution factor. Homogenate was measured for 29 common cytokines with MSD 29-plex ELISA kit (K15267G, Meso Scale Development, Rockville, MD, United States). Data were read on the MSD QuickPlex 120.

### MicroCT

A harvest technique optimized for MicroCT was utilized in order to ensure maximal perfusion of the vascular space (Ghanavati et al., 2014). Animals were terminally anesthetized with 5% isoflurane, and transcardially perfused with 20 ml warm heparinized PBS, followed by 20 ml microfill solution MV-122 (MicroCT, San Francisco, CA, United States), mixed immediately before infusion. Fluids were infused at a rate of 2 ml/min. Brains were collected and fixed in 10% formalin solution for a minimum of 24 h, prior to scanning. Fixed brains were then scanned using the Skyscan 1172, 12-megapixel, high-resolution cone-beam microCT scanner (Bruker, Kontich, Belgium). Scan parameters involved using an isotropic voxel size of 3.0 μm, a source voltage of 100 kV, and a current of 100 μA. Following scanning, 3D reconstruction was performed using Skyscan N-recon software (Micro Photonics, Inc., Allentown, PA, United States). The vascular spaces were reconstructed at a resolution of 0.5 uM/pixel (Quintana et al., 2019) and analyzed for mean vessel thickness, overall perfusion, and number of vascular leakages (Quintana et al., 2019). All procedures were conducted in accordance with standardized methods for rodent tissue (Dyer et al., 2017; Karreman et al., 2017; Quintana et al., 2019).

### miRNA Functional Analysis

The mature sequences of the miRNAs were retrieved using miRBase database: mu-mir-98 no. MIMAT0000096:UGAGGUAGUAAGUUGUAUUGUU and mu-let-7g* no. MIMAT0004584:CUGUACAGGCCACUGCCU- UGC and were synthetized by Integrated DNA Technologies, Inc. (IDT, Coralville, IA, United States). The IP-10 3′UTR and CXCL1 3′UTR sequences, cloned downstream to the firefly luciferase sequence in the pMirTarget reporter vector (further pMir), were purchased from OriGene (OriGene Technologies, Inc., Rockville, MD, United States). For the perfect match sequence, the mature sequence of mu-miR-98 or mu-let-7g* synthetic oligos was transfected together with pMir reporter plasmids, containing the corresponding 3′UTRs.

To confirm specificity of miR-mRNA binding to target 3′UTR, the miR’s seed-binding sequence was mutated in each of the 3′ UTRs (marked in bold and underlined in Figure 2, the nucleotides were changed for complementary one). The IP-10 3′UTR mutated in the mir-98 or let-7g* seeding sequence was generated by site directed mutagenesis (Agilent Technologies, Santa Clara, CA, United States) using the pMir/IP-10 3′UTR as a template. The oligonucleotides for the mutagenesis were as follows: forward, 5′-GGACCACACAGAGGC**ACGG**TCT (mutated bases in the mir-98 seeding sequence are in bold and underlined) and 5′-CCCAAATTCTTTCAGT**CCGA**ACCTAC (mutated bases in the let-7g* seeding sequence are in bold and underlined). The CXCL1 3′UTR mutated in the mir-98 or let-7g* seeding sequence was generated by site directed mutagenesis (Agilent) using the pMir/CXCL1 3′UTR as a template. The oligonucleotides for the mutagenesis were as follows: forward, 5′-GATGGGTAGGCTTAAAATA**AAAG**AT (mutated bases in the mir-98 seeding sequence are in bold and underlined) and 5′-GGAGGCTGTGT**AACA**ATG (mutated bases in the let-7g* seeding sequence are in bold and underlined). The reverse primers were complementary to the forward for all mentioned above sequences. All primers were synthetized by IDT. *Caenorhabditis elegans* miR-39 (cel-39), MIMAT0020306:AGCUGAUUUCGUCUUGGUAAUA was synthetized by IDT and was used as a non-specific/non-targeting control (Rom et al., 2015; Bernstein et al., 2019, 2020).

### Luciferase Assay

For miR target validation, HEK 293 cells were plated at a concentration of 8 × 10^4^ cells/well in a 12-well plate in DMEM with 10% FBS medium. The following day, a total amount of 0.5 μg DNA/well was transfected utilizing Lipofectamine (Life Technologies) at a DNA:Lipofectamine ratio of 1:3. pcDNA3 was added to keep the total amount of DNA constant. Samples were harvested 48 h post-transfection and subjected to the luciferase assay system (Promega, Madison, WI, United States) following the manufacturer’s instructions using a Infinity M200PRO chemiluminometer (Tecan Group Ltd., Mannedof, Switzerland). Relative units represent the ratio between luciferase values of the sample and the non-targeting control. The experiments were performed in duplicate and repeated at least three times (Rom et al., 2015), averaged and each mean shown in the graph as one point.

### Statistical Analysis

Data are expressed as the mean ± SD of experiments conducted multiple times. Data were tested for normality using the Shapiro–Wilk test, and, if data were normally distributed, for multiple group comparisons. Multiple group comparisons were performed by one-way ANOVA with Tukey *post hoc* test with significance at *p* < 0.05. A paired two-tailed Student’s test was used to compare before and after effects. Significant differences were considered to be at *p* < 0.05. Statistical analyses were performed utilizing Prism v8 software (GraphPad Software Inc., San Diego, CA, United States). To determine the number of samples used for quantitative assays, a power calculation was performed based on the expected variability between testing conditions using the following equation: *n* = 1 + 2C${\left(\frac{s}{d}\right)}^{2}$, where C is a constant equal to 10.51 for a power of 90% and a confidence interval of 95%, *s* is the variance, and *d* is the difference between conditions. We determined optimal sample sizes by calculating the number of animals required to produce an *N* of sufficient size to perform one-way ANOVA analysis of a standard distribution, which we determined to be 4, and 3 animals analyzed in duplicates would provide at least six data points for each experiment. For ELISA and microCT, all samples were run in duplicate and combined to produce a weighted average value. Each calculation is represented by one data point. For reporter assays, experiments were repeated twice in triplicate with average value of both replicates used as a single data point.

## Results

### Let-7g* and miR-98 Reduce Stroke-Induced Production of Proinflammatory Cytokines in Mouse Brain

**FIGURE 1:**
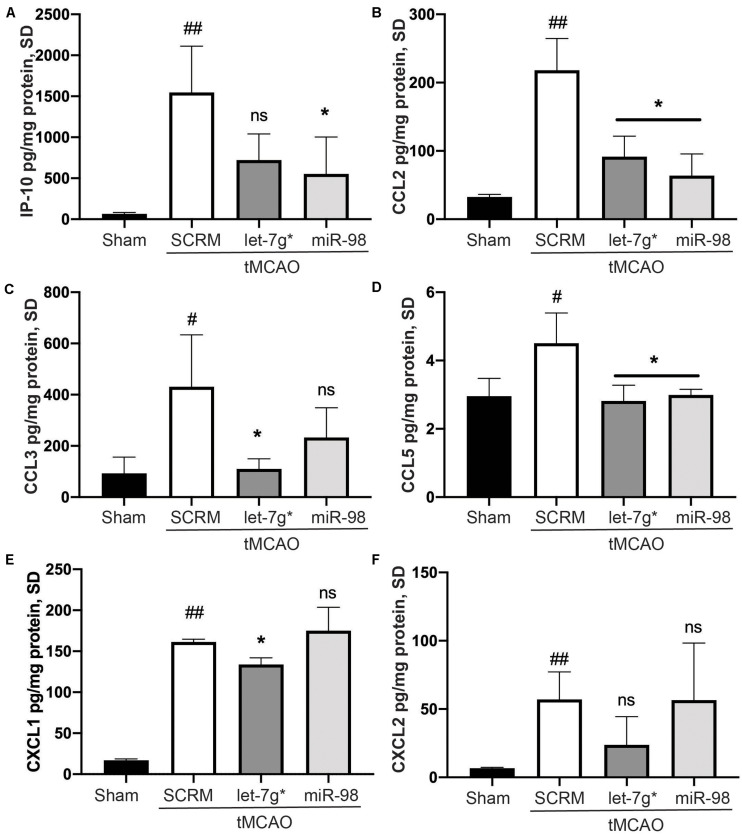
Let-7 miRs differentially reduce cytokine release following stroke. ELISA analysis of IP-10 **(A)**, CCL2 **(B)**, CCL3 **(C)**, CCL5 **(D)**, CXCL2 **(E)**, and KC/GRO (CXCL-1 mouse analog) **(F)** expression. Mice were subjected to 60 min ischemia and 72 h reperfusion performed as described in section “Materials and Methods.” Brains were harvested following anesthesia, homogenized, and were used to run all assays. Results are presented as mean ± SD from at least two independent experiments (*n* = 4). #*p* < 0.05 (SCRM); *p < 0.05 (let-7g*, miR-98). ##*p* < 0.01 (SCRM); ns is not significant.

In recent studies, we have shown that both let-7g* and miR-98 exert anti-inflammatory impacts on endothelial cells within the BBB (Rom et al., 2015; Bernstein et al., 2019, 2020) and reduce the damage caused by ischemic stroke. However, these miRs may produce differing effects on the specific nature of immune cell infiltration into the brain parenchyma following stroke. To determine which inflammatory signals were reduced by let-7 miRs, we measured the expression of 29 different cytokines from the homogenate of the stroke-infarcted hemisphere. We determined that tMCAO significantly increased the expression of IP-10 (*p* < 0.01), CXCL1 (*p* < 0.005), CXCL2 (*p* < 0.01), CCL2 (*p* < 0.01), CCL3 (*p* < 0.05), and CCL5 (*p* < 0.05) compared with baseline (Figures 1A–F). Both miRs significantly reduced the stroke-induced increase in CCL2 (*p* < 0.05) and CCL5 (*p* < 0.01; Figures 1B,D). However, only let-7g* was effective in reducing the stroke-driven increases in CCL3 (*p* < 0.05) and CXCL1 (*p* < 0.005; Figures 1C,F), while miR-98 attenuated the increase in IP-10 (*p* < 0.05) only (Figure 1A).

### miR-98 Specifically Targets IP-10 mRNA, While Let-7g* Targets IL-8-Mouse Homolog KC/GRO (CXCL1)

**FIGURE 2:**
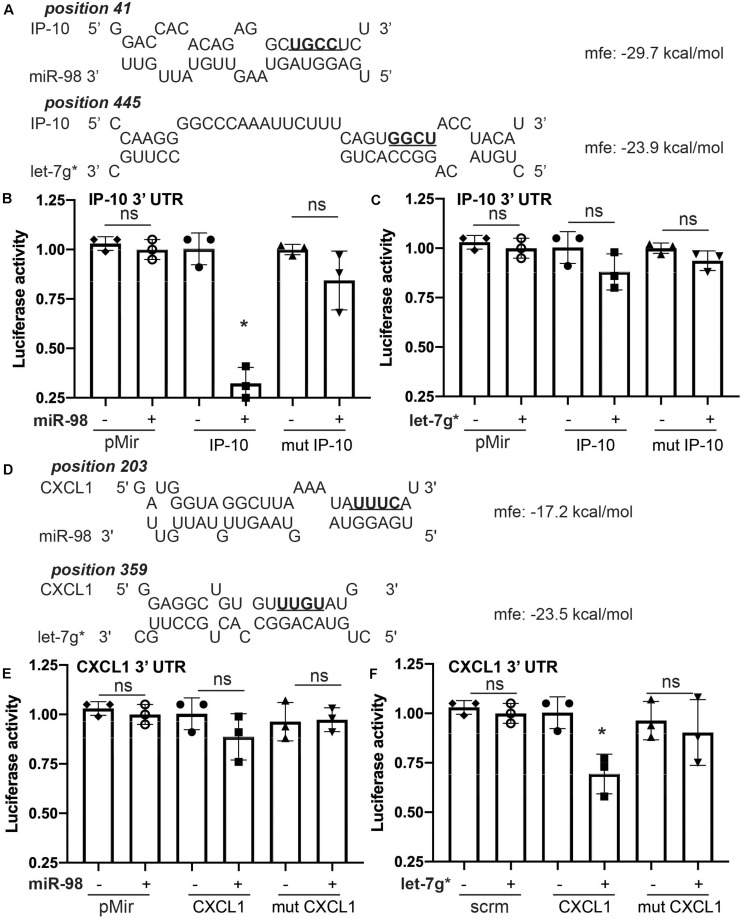
Let-7g* and miR-98 selectively target different cytokine mRNAs. Prediction analysis for miR-98 and let-7g* for the ability to create miR-mRNA hybrid with IP-10 **(A)** and CXCL1 **(D)** 3′ UTRs, with minimum free energy recorded. Luciferase activity for IP-10′s 3′ UTR reporter in HEK-293 cells transfected with mimic oligos of miR-98 **(B)** or let-7g* **(C)**. Luciferase activity for CXCL1’s 3′ UTR reporter with mimic oligos of miR-98 **(E)** or let-7g* **(F)**. 3′ UTR sequences of IP-10 and CXCL1 were mutated in the seed-binding site (underlined and bolded). Data are shown as mean ± SD. *p < 0.05.

Sequence complementarity is the most critical measure in the relative power of miRNAs to silence their mRNA targets. Despite the importance of strong seed binding, extensive downstream (toward the 3′ end) pairing can sometimes compensate for imperfect seed binding (Shin et al., 2010). To estimate binding affinity of the let-7 miRs of interest, we used the RNAhybrid sequence tool (Bibiserv, Bielefeld, Germany; Rehmsmeier et al., 2004) to predict the miRNA-mRNA hybridization of let-7g* and miR-98 with the 3′ UTR sequences of several cytokines, identified in our ELISA screen (Figure 1). We found substantial complementarity between let-7g* seed with IL-8-mouse homolog KC/GRO (CXCL1)′ 3′ UTR, yielding a minimum free energy of -23.5 kcal/mol. By contrast, miR-98 showed a lower binding affinity, resulting in a minimum free energy of 17.2 kcal/mol (Figure 2D). In addition, miR-98 was shown to have higher binding affinity with IP-10 3′ UTR, with a minimum free energy of −29.7 kcal/mol (Figure 2A). let-7g* also showed some degree of bonding affinity (mfe = −23.9 kcal/mol) with IP-10 3′ UTR sequence, but the complementarity was not significant (Figure 2B), due to imperfect binding in the seed region.

To confirm this binding affinity, we performed the dual-luciferase assay in 293 HEK cells following a standard transfection protocol^8^. Cells were transfected with plasmids containing wild-type (WT) or mutated 3′UTR of IP-10 or IL-8-mouse homolog CXCL1′ fused with luciferase reporter and co-transfected with miR-98 and let-7g* mimic miRNA sequences. Mutated 3′ UTRs contained four nucleotides switched within the miR-seed binding region (Figures 2A,D, in bold). miR-98 downregulated the activity of WT IP-10 3′UTR-reporter by nearly 3.1-fold (*p* < 0.05; Figure 2B), while in the mutated 3′UTR-reporter, miR-98 co-transfection induced almost no effect. By contrast, let-7g* produced a much more modest, statistically insignificant decline in WT IP-10 3′UTR-reporter activity, a luciferase levels of mutated IP-10 3′UTR-reporter activity was not much different from that produced in WT (Figure 2C). With WT IL-8 3′UTR, the impacts of the miRNAs were inverted. let-7g* reduced activity by 1.5-fold (*p* < 0.05) in WT IL-8 3′UTR, while in the mutated IL-8 3′UTR reporter, let-7g* did not alter activity significantly. Similar results were obtained when cells containing cloned IP-10 or IL-8-mouse homolog CXCL1′ 3′UTR fused with luciferase reporter were transfected with normal or mutated forms of miR-98 and let-7g* mimic miRNA sequences (Supplementary Figure 1). Mutated forms of miRs contained four nucleotides switched within the seed region (Supplementary Figures 1A,B, in bold). Normal miR-98 downregulated the activity of IP-10 3′UTR-reporter by nearly 77% ± 14% (*p* < 0.05; Supplementary Figure 1C), while the mutated form induced almost no effect. With IL-8 (CXCL1) 3′UTR, wild type let-7g* oligo reduced activity by 48% ± 7.5% (*p* < 0.05), while the mutated form did not alter activity significantly (Supplementary Figure 1D). This research expands significantly upon existing research regarding the regulation of cytokines by let-7 miRs. We have previously shown how let-7g* and miR-98 demonstrate strong seed binding to and inhibition of both CCL2 and CCL5 (Rom et al., 2015). In the same paper, we show that such impacts can strongly influence the degree of leukocyte adhesion to endothelial cells during inflammation. Together, our findings illustrate the importance of let-7 miRs in regulating the immune response to neurovascular insult.

### Let-7g* Significantly Reduces Leakage and Improves Vascular Function After Stroke

**FIGURE 3:**
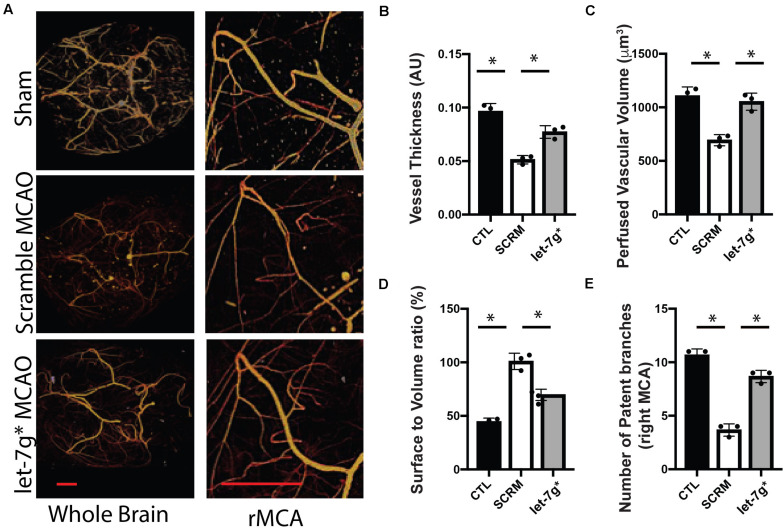
let-7g* attenuates vascular damage after tMCAO. Cerebral neurovasculature following sham control or stroke was imaged with MicroCT, as detailed in section “Materials and Methods” **(A)**. In right (tMCAO) hemisphere, the mean perfused vessel thickness was measured relative to control hemisphere **(B)**, total volume of vascularized brain tissue **(C)**, vascular surface area relative to perfused volume **(D)**, and the number of patent branches originating from the right middle cerebral artery **(E)**. Data are shown as mean ± SD. ^∗^*p* < 0.05. Scale bar = 1 mm.

Recent studies have denoted that restoration of let-7 miRNA levels may be strongly neuroprotective, particularly after significant inflammatory insults that occur following ischemic stroke (Li et al., 2019; Bernstein et al., 2020). We have previously shown that such treatments reduce the permeability toward both large (>10 kD) molecules, and multiple types of immune cells (Bernstein et al., 2019, 2020). To fully assess the impact upon the BBB, post-MCAO animals were perfused with Microfil, and the neurovasculature was mapped using X-ray tomography, with an enhanced focus on the MCA (Figure 3A). We determined that tMCAO reduced the average diameter of the MCA to ∼50% the size in control animals (*p* < 0.01), while treatment with let-7g* attenuated this decrease by nearly 60% (Figures 3A,B; *p* < 0.05). Further, injection of let-7g* prevented a significant degree of the stroke-induced reduction of perfused vascular volume (Figure 3C), restored vessel volume-surface ratio to nearly baseline levels (*p* = 0.69) (Figure 3D), and prevented the loss of seven of nine major arterial branches on the MCA (Figure 3E).

Taken together with our previously published data, we conclude that let-7g* and miR-98 diminish stroke-induced increase in CXCL1 and IP-10 protein levels, respectively, by direct targeting their 3′ UTR sequence. Both miRs, let-7g* and miR-98 directly target CCL2 and CCL5 cytokines and regulate their expression (Rom et al., 2015). These effects on cytokine expression allow let-7 miRs to preserve the cerebral vasculature following tMCAO, exhibited by attenuating the stroke-induced reduction of vessel thickness, perfused brain volume, and number of patent arteries originating at the MCA, and by lessening the stroke-induced spike in vascular surface/volume ratio. These findings underscore the role of let-7g* overexpression in maintaining vascular homeostasis following inflammatory insult.

## Discussion

We have previously demonstrated how let-7 miRs preserve the integrity of the BBB, reduce cytokine release *in vitro*, and inhibit recruitment of pro-inflammatory immune cells from both sides of the BBB, leading to better functional recovery. In this study, we show how miR-98 and let-7g* confer such neuroprotection through slightly different mechanisms. The current research denotes that let-7g* attenuates CXCL1 (IL-8-mouse homolog), which can lower CXCR2 activity, which is critical for recruiting neutrophils (Easton, 2013; Giles et al., 2018; Bernstein et al., 2020). This mechanism is likely responsible for a significant degree of let-7g*-induced neuroprotection; IL-8 is directly linked to endothelial activation and leukocyte recruitment (Wu et al., 2015), and silencing its activity in endothelial cells strongly decreases inflammation-induced permeability (Chen et al., 2018). Increased IL-8 binding to CXCR2 has been shown to reduce vascular wall thickness (Varney et al., 2006), which can worsen hypertension decrease in the area covered by the vasculature (Wang et al., 2016). As MicroCT scanning illustrated how let-7g* increases vascular thickness, vessel volume, and patent arterial branches, and previous work that indicated that let-7g* reduces the number of IL-8-recruited cells into the penumbra (Bernstein et al., 2020), we find it highly plausible that let-7g*-induced neuroprotection involves IL-8 mediated changes within the neurovasculature. The let-7g*-induced neuroprotection may stem from binding to the 3′ UTR of CXCR2 mRNA through targeting of its transcriptional regulators such as NF-kB and CREB (Amiri and Richmond, 2003), or a combination.

Conversely, the let-7 miR family member, miR-98, was shown to bind to and strongly reduce IP-10, which corresponds to the let-7-specific reduction of brain-infiltrating T-cells (Bernstein et al., 2019). These findings are in line with current research which indicates let-7s are critical for mediating endothelial-T cell interactions, and may exert a different effect on the neurovasculature, though some elements are common to both miRs. miRs from the let-7 family directly diminish the expression of MCP-1/CCL2 and RANTES/CCL5 cytokines (Rom et al., 2015), which are complicated in the progression of BBB inflammation that happens during traumatic brain injury (Lumpkins et al., 2008; Albert et al., 2017), various forms of encephalitis (Chowdhury and Khan, 2017) and diabetes (Zhang, 2008; Teler et al., 2017; Lee et al., 2019). Although there are numerous triggers for neuroinflammation, most of them include amplified levels of these particular cytokines, which can cause leukocyte adhesion (Schober, 2008) and rearrangement of tight junction proteins on endothelial cells (Stamatovic et al., 2005), leading to BBB compromise. BBB disruption, upregulation of cell adhesion molecules, and activation of resident microglia develop the post-stroke neuro-immune interactions. This report found that only let-7g* was effective in reducing the stroke-driven increases in CCL3, whereas miR-98 did not affect its expression. CCL3 cytokine has been implicated in the enhancement of BBB permeability and the reduction of TJ protein expression (Chai et al., 2014). Whether CCL3 reduction was due to direct or indirect let-7g* miR targeting should be investigated in future studies.

**FIGURE 4:**
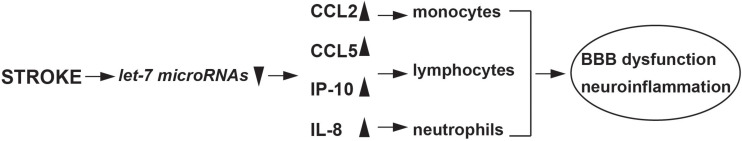
Schematic of stroke induced let-7 miRNAs changes and immune response.

Previous research has shown how let-7g* and miR-98 can both activate the innate immune response, and this is corroborated by current findings, which indicate pronounced effects on CCL5 and CCL2 release (Rom et al., 2015), corresponding to reduced monocyte brain infiltration, macrophage, and microglial activation (Bernstein et al., 2019, 2020). This research adds to the growing pool of evidence on the diverse neurovascular impacts of the let-7 family (Figure 4). For instance, let-7a can inhibit the proinflammatory response in microglia, by lowering nitric oxide signaling and altering the pattern of cytokine release (Cho et al., 2015), and let-7 derived from exosomes can strongly inhibit atherosclerotic inflammation via the PTEN pathway (Li et al., 2019). However, not all miRs from the let-7 family produce beneficial effects to the vascular space. For instance, let-7f is capable of worsening the effects of stroke, by inhibiting insulin-like growth factor 1 (IGF-1) signaling (Selvamani et al., 2012). Further, let-7a, b, e, and f have been postulated to exacerbate neuronal damage following inflammation through actions on toll-like receptor 7 (Mueller et al., 2014). With such varied results, it is critical to understand the mechanisms through which let-7 miRs influence the vascular space and when their use might be contraindicated. The BBB can respond to inflammatory stimuli in multiple ways, and understanding the role of miRs in mediating such responses are critical for developing effective treatments for stroke. Combinatorial approaches have been suggested, which utilize multiple miRs from the same family (Khoshnam et al., 2017). This is an interesting approach, and one well-supported by current siRNA research, which suggests that multiple combinations of similar siRNAs can be more effective than a higher concentration of a single miRNA (Bahi et al., 2005). While further experiments are needed to fully elucidate the nature of let-7 impacts on ischemic stroke, studies such as this one are critical for understanding the role of miRs in mediating inflammatory damage, and will be critical for developing more effective treatments.

We found that changes in miRNA expression seen in primary human BMVEC *in vitro* occur *in vivo* in an animal model of neuroinflammation (Rom et al., 2015) and in a stroke tMCAO model (Bernstein et al., 2019, 2020). Overexpression of miR-98 and let-7g* in brain endothelium attenuated leukocyte adhesion/migration and diminished BBB permeability pointing to functional reproducibility of miRNA effects *in vitro* systems. We have previously established that let-7 miRs preserve the integrity of the BBB, diminish cytokine release *in vitro* (Rom et al., 2015), and reduce recruitment of pro-inflammatory immune cells from blood to CNS *in vivo*, leading to better functional recovery (Bernstein et al., 2019, 2020), corroborating that let-7 miRNAs have remedial potential in neuroinflammation. In the current study, we focused our analysis on the mechanisms by which miR-98 and let-7g* overexpression impacts cytokine expression following ischemic stroke and evaluated their impacts upon the neurovascular structure.

In summary, using a functional approach, we have identified a mechanism implicated in the negative regulation of inflammation in endothelium and brain after stroke. It involves the stroke-mediated decrease of miR-98 and let-7g*, which in turn, triggers expression of pro-inflammatory mediators, such as CCL2, CCL5, CXCL1, and IP-10. Our data support a role for let-7 miRNAs in modulation of inflammatory processes in stroke-induced inflammation and preserve the cerebral vasculature following tMCAO.

## Data Availability Statement

The original contributions presented in the study are included in the article/Supplementary Material, further inquiries can be directed to the corresponding author.

## Ethics Statement

The animal study was reviewed and approved by Temple University Institutional Animal Care and Use Committee.

## Author Contributions

DB: data acquisition and analysis, drafting and revising, and final approval. SR: conception and design, data acquisition, analysis and interpretation, drafting and revising the article, and final approval.

## Conflict of Interest

The authors declare that the research was conducted in the absence of any commercial or financial relationships that could be construed as a potential conflict of interest.

## Footnotes

1 www.nc3rs.org.uk/arrive-guidelines

## References

[B1] Ahnstedt H. Sweet J. Cruden P. Bishop N. Cipolla M. J. (2016). Effects of early post-ischemic reperfusion and tPA on Cerebrovascular function and nitrosative stress in female rats. *Transl. Stroke Res.* 7 228–238. 10.1007/s12975-016-0468-4 27125535 PMC5291115

[B2] Albert V. Subramanian A. Agrawal D. Bhoi S. K. Pallavi P. Mukhopadhayay A. K. (2017). RANTES levels in peripheral blood, CSF and contused brain tissue as a marker for outcome in traumatic brain injury (TBI) patients. *BMC Res. Notes* 10:139. 10.1186/s13104-017-2459-2 28340601 PMC5366123

[B3] Amiri K. I. Richmond A. (2003). Fine tuning the transcriptional regulation of the CXCL1 chemokine. *Prog. Nucleic Acid Res. Mol. Biol.* 74 1–36. 10.1016/s0079-6603(03)01009-2 14510072 PMC3140403

[B4] Bahi A. Boyer F. Kolira M. Dreyer J. L. (2005). In vivo gene silencing of CD81 by lentiviral expression of small interference RNAs suppresses cocaine-induced behaviour. *J. Neurochem.* 92 1243–1255. 10.1111/j.1471-4159.2004.02961.x 15715673

[B5] Bernstein D. L. Gajghate S. Reichenbach N. L. Winfield M. Persidsky Y. Heldt N. A. (2020). let-7g counteracts endothelial dysfunction and ameliorating neurological functions in mouse ischemia/reperfusion stroke model. *Brain Behav. Immun.* 87 543–555. 10.1016/j.bbi.2020.01.026 32017988 PMC7316629

[B6] Bernstein D. L. Zuluaga-Ramirez V. Gajghate S. Reichenbach N. L. Polyak B. Persidsky Y. (2019). miR-98 reduces endothelial dysfunction by protecting blood-brain barrier (BBB) and improves neurological outcomes in mouse ischemia/reperfusion stroke model. *J. Cereb. Blood Flow Metab.* 10.1177/0271678x19882264 [Epub ahead of print] 31601141 PMC7786850

[B7] Chai Q. He W. Q. Zhou M. Lu H. Fu Z. F. (2014). Enhancement of blood-brain barrier permeability and reduction of tight junction protein expression are modulated by chemokines/cytokines induced by rabies virus infection. *J. Virol.* 88 4698–4710. 10.1128/jvi.03149-13 24522913 PMC3993813

[B8] Chen Q. F. Liu Y. Y. Pan C. S. Fan J. Y. Yan L. Hu B. H. (2018). Angioedema and Hemorrhage After 4.5-Hour tPA (Tissue-Type Plasminogen Activator) Thrombolysis Ameliorated by T541 via Restoring Brain Microvascular Integrity. *Stroke* 49 2211–2219. 10.1161/strokeaha.118.021754 30354988

[B9] Cho K. J. Song J. Oh Y. Lee J. E. (2015). MicroRNA-Let-7a regulates the function of microglia in inflammation. *Mol. Cell Neurosci.* 68 167–176. 10.1016/j.mcn.2015.07.004 26221772

[B10] Chowdhury P. Khan S. A. (2017). Significance of CCL2, CCL5 and CCR2 polymorphisms for adverse prognosis of Japanese encephalitis from an endemic population of India. *Sci. Rep.* 7:13716. 10.1038/s41598-017-14091-8 29057937 PMC5651904

[B11] Dimitrijevic O. B. Stamatovic S. M. Keep R. F. Andjelkovic A. V. (2006). Effects of the chemokine CCL2 on blood-brain barrier permeability during ischemia-reperfusion injury. *J. Cereb. Blood Flow Metab.* 26 797–810. 10.1038/sj.jcbfm.9600229 16192992

[B12] Dyer E. L. Gray Roncal W. Prasad J. A. Fernandes H. L. Gursoy D. De Andrade V. (2017). Quantifying Mesoscale Neuroanatomy Using X-Ray Microtomography. *eNeuro* 4:e0195-17. 10.1523/eneuro.0195-17.2017 29085899 PMC5659258

[B13] Easton A. S. (2013). Neutrophils and stroke - can neutrophils mitigate disease in the central nervous system? *Int. Immunopharmacol.* 17 1218–1225. 10.1016/j.intimp.2013.06.015 23827753

[B14] Engel O. Kolodziej S. Dirnagl U. Prinz V. (2011). Modeling stroke in mice - middle cerebral artery occlusion with the filament model. *J. Vis. Exp.* 47:2423. 10.3791/2423 21248698 PMC3182649

[B15] Ghanavati S. Yu L. X. Lerch J. P. Sled J. G. (2014). A perfusion procedure for imaging of the mouse cerebral vasculature by X-ray micro-CT. *J. Neurosci. Methods* 221 70–77. 10.1016/j.jneumeth.2013.09.002 24056228

[B16] Giles J. A. Greenhalgh A. D. Denes A. Nieswandt B. Coutts G. McColl B. W. (2018). Neutrophil infiltration to the brain is platelet-dependent, and is reversed by blockade of platelet GPIbalpha. *Immunology* 154 322–328. 10.1111/imm.12892 29325217 PMC5979746

[B17] Hou W. Tian Q. Steuerwald N. M. Schrum L. W. Bonkovsky H. L. (2012). The let-7 microRNA enhances heme oxygenase-1 by suppressing Bach1 and attenuates oxidant injury in human hepatocytes. *Biochim. Biophys. Acta* 1819 1113–1122. 10.1016/j.bbagrm.2012.06.001 22698995 PMC3480558

[B18] Huang H. C. Yu H. R. Hsu T. Y. Chen I. L. Huang H. C. Chang J. C. (2017). MicroRNA-142-3p and let-7g Negatively Regulates Augmented IL-6 Production in Neonatal Polymorphonuclear Leukocytes. *Int. J. Biol. Sci.* 13 690–700. 10.7150/ijbs.17030 28655995 PMC5485625

[B19] Jickling G. C. Ander B. P. Shroff N. Orantia M. Stamova B. Dykstra-Aiello C. (2016). Leukocyte response is regulated by microRNA let7i in patients with acute ischemic stroke. *Neurology* 87 2198–2205. 10.1212/wnl.0000000000003354 27784773 PMC5123554

[B20] Jin R. Yang G. Li G. (2010a). Inflammatory mechanisms in ischemic stroke: role of inflammatory cells. *J. Leukoc Biol.* 87 779–789. 10.1189/jlb.1109766 20130219 PMC2858674

[B21] Jin R. Yang G. Li G. (2010b). Molecular insights and therapeutic targets for blood-brain barrier disruption in ischemic stroke: critical role of matrix metalloproteinases and tissue-type plasminogen activator. *Neurobiol. Dis.* 38 376–385. 10.1016/j.nbd.2010.03.008 20302940 PMC2862862

[B22] Jolana L. Kamil D. (2017). The role of microRNA in ischemic and hemorrhagic stroke. *Curr. Drug Deliv.* 14 816–831. 10.2174/1567201813666160919142212 27691978

[B23] Joshi S. Wei J. Bishopric N. H. (2016). A cardiac myocyte-restricted Lin28/let-7 regulatory axis promotes hypoxia-mediated apoptosis by inducing the AKT signaling suppressor PIK3IP1. *Biochim. Biophys. Acta* 1862 240–251. 10.1016/j.bbadis.2015.12.004 26655604 PMC4732518

[B24] Karreman M. A. Ruthensteiner B. Mercier L. Schieber N. L. Solecki G. Winkler F. (2017). Find your way with X-Ray: using microCT to correlate in vivo imaging with 3D electron microscopy. *Methods Cell Biol.* 140 277–301. 10.1016/bs.mcb.2017.03.006 28528637

[B25] Khoshnam S. E. Winlow W. Farbood Y. Moghaddam H. F. Farzaneh M. (2017). Emerging Roles of microRNAs in ischemic stroke: as possible therapeutic agents. *J. Stroke* 19 166–187. 10.5853/jos.2016.01368 28480877 PMC5466283

[B26] Lambertsen K. L. Biber K. Finsen B. (2012). Inflammatory cytokines in experimental and human stroke. *J. Cereb. Blood Flow Metab.* 32 1677–1698. 10.1038/jcbfm.2012.88 22739623 PMC3434626

[B27] Lee C. P. Nithiyanantham S. Hsu H. T. Yeh K. T. Kuo T. M. Ko Y. C. (2019). ALPK1 regulates streptozotocin-induced nephropathy through CCL2 and CCL5 expressions. *J. Cell Mol. Med.* 23 7699–7708. 10.1111/jcmm.14643 31557402 PMC6815771

[B28] Li H. W. Meng Y. Xie Q. Yi W. J. Lai X. L. Bian Q. (2015). miR-98 protects endothelial cells against hypoxia/reoxygenation induced-apoptosis by targeting caspase-3. *Biochem. Biophys. Res. Commun.* 467 595–601. 10.1016/j.bbrc.2015.09.058 26367177

[B29] Li S. Chen L. Zhou X. Li J. Liu J. (2019). miRNA-223-3p and let-7b-3p as potential blood biomarkers associated with the ischemic penumbra in rats. *Acta Neurobiol. Exp.* 79 205–216. 10.21307/ane-2019-018 31342956

[B30] Li W. Pan R. Qi Z. Liu K. J. (2018). Current progress in searching for clinically useful biomarkers of blood-brain barrier damage following cerebral ischemia. *Brain Circ.* 4 145–152. 10.4103/bc.bc_11_18 30693340 PMC6329218

[B31] Liu M. Tao G. Liu Q. Liu K. Yang X. (2017). MicroRNA let-7g alleviates atherosclerosis via the targeting of LOX-1 in vitro and in vivo. *Int. J. Mol. Med.* 40 57–64. 10.3892/ijmm.2017.2995 28535009 PMC5466378

[B32] Liu Y. Chen Q. Song Y. Lai L. Wang J. Yu H. (2011). MicroRNA-98 negatively regulates IL-10 production and endotoxin tolerance in macrophages after LPS stimulation. *FEBS Lett.* 585 1963–1968. 10.1016/j.febslet.2011.05.029 21609717

[B33] Lumpkins K. Bochicchio G. V. Zagol B. Ulloa K. Simard J. M. Schaub S. (2008). Plasma levels of the beta chemokine regulated upon activation, normal T cell expressed, and secreted (RANTES) correlate with severe brain injury. *J. Trauma* 64 358–361. 10.1097/TA.0b013e318160df9b 18301198

[B34] Mueller M. Zhou J. Yang L. Gao Y. Wu F. Schoeberlein A. (2014). PreImplantation factor promotes neuroprotection by targeting microRNA let-7. *Proc. Natl. Acad. Sci. U.S.A.* 111 13882–13887. 10.1073/pnas.1411674111 25205808 PMC4183321

[B35] Nayak A. R. Kashyap R. S. Kabra D. Purohit H. J. Taori G. M. Daginawala H. F. (2012). Time course of inflammatory cytokines in acute ischemic stroke patients and their relation to inter-alfa trypsin inhibitor heavy chain 4 and outcome. *Ann. Indian Acad. Neurol.* 15 181–185. 10.4103/0972-2327.99707 22919189 PMC3424794

[B36] Nour M. Scalzo F. Liebeskind D. S. (2013). Ischemia-reperfusion injury in stroke. *Interv. Neurol.* 1 185–199. 10.1159/000353125 25187778 PMC4031777

[B37] Pawluk H. Wozniak A. Grzesk G. Kolodziejska R. Kozakiewicz M. Kopkowska E. (2020). The role of selected pro-inflammatory cytokines in pathogenesis of ischemic stroke. *Clin. Interv. Aging* 15 469–484. 10.2147/cia.S233909 32273689 PMC7110925

[B38] Quintana D. D. Lewis S. E. Anantula Y. Garcia J. A. Sarkar S. N. Cavendish J. Z. (2019). The cerebral angiome: high resolution MicroCT imaging of the whole brain cerebrovasculature in female and male mice. *Neuroimage* 202 116109. 10.1016/j.neuroimage.2019.116109 31446129 PMC6942880

[B39] Rehmsmeier M. Steffen P. Hochsmann M. Giegerich R. (2004). Fast and effective prediction of microRNA/target duplexes. *RNA* 10 1507–1517. 10.1261/rna.5248604 15383676 PMC1370637

[B40] Rom S. Dykstra H. Zuluaga-Ramirez V. Reichenbach N. L. Persidsky Y. (2015). miR-98 and let-7g* protect the blood-brain barrier under neuroinflammatory conditions. *J. Cereb. Blood Flow Metab.* 35 1957–1965. 10.1038/jcbfm.2015.154 26126865 PMC4671116

[B41] Schober A. (2008). Chemokines in vascular dysfunction and remodeling. *Arterioscler Thromb. Vasc. Biol.* 28 1950–1959. 10.1161/ATVBAHA.107.161224 18818421

[B42] Selvamani A. Sathyan P. Miranda R. C. Sohrabji F. (2012). An antagomir to microRNA Let7f promotes neuroprotection in an ischemic stroke model. *PLoS One* 7:e32662. 10.1371/journal.pone.0032662 22393433 PMC3290559

[B43] Sen C. K. Gordillo G. M. Khanna S. Roy S. (2009). Micromanaging vascular biology: tiny microRNAs play big band. *J. Vasc. Res.* 46 527–540. 10.1159/000226221 19571573 PMC2803349

[B44] Shin C. Nam J. W. Farh K. K. Chiang H. R. Shkumatava A. Bartel D. P. (2010). Expanding the microRNA targeting code: functional sites with centered pairing. *Mol. Cell* 38 789–802. 10.1016/j.molcel.2010.06.005 20620952 PMC2942757

[B45] Stamatovic S. M. Shakui P. Keep R. F. Moore B. B. Kunkel S. L. Van Rooijen N. (2005). Monocyte chemoattractant protein-1 regulation of blood-brain barrier permeability. *J. Cereb. Blood Flow Metab.* 25 593–606. 10.1038/sj.jcbfm.9600055 15689955

[B46] Tarkowski E. Rosengren L. Blomstrand C. Wikkelso C. Jensen C. Ekholm S. (1997). Intrathecal release of pro- and anti-inflammatory cytokines during stroke. *Clin. Exp. Immunol.* 110 492–499. 10.1046/j.1365-2249.1997.4621483.x 9409656 PMC1904815

[B47] Teler J. Tarnowski M. Safranow K. Maciejewska A. Sawczuk M. Dziedziejko V. (2017). CCL2, CCL5, IL4 and IL15 gene polymorphisms in women with gestational diabetes mellitus. *Horm. Metab. Res.* 49 10–15. 10.1055/s-0042-111436 27472286

[B48] Varney M. L. Johansson S. L. Singh R. K. (2006). Distinct expression of CXCL8 and its receptors CXCR1 and CXCR2 and their association with vessel density and aggressiveness in malignant melanoma. *Am. J. Clin. Pathol.* 125 209–216. 10.1309/vpl5-r3jr-7f1d-6v03 16393674

[B49] Wang G. Zhang Z. Ayala C. Dunet D. O. Fang J. George M. G. (2014). Costs of hospitalization for stroke patients aged 18-64 years in the United States. *J. Stroke Cerebrovasc. Dis.* 23 861–868. 10.1016/j.jstrokecerebrovasdis.2013.07.017 23954598 PMC4544732

[B50] Wang L. Zhao X. C. Cui W. Ma Y. Q. Ren H. L. Zhou X. (2016). Genetic and pharmacologic inhibition of the chemokine receptor CXCR2 prevents experimental hypertension and vascular dysfunction. *Circulation* 134 1353–1368. 10.1161/circulationaha.115.020754 27678262 PMC5084654

[B51] Weng Z. Zhao Q. (2015). Utilizing ELISA to monitor protein-protein interaction. *Methods Mol. Biol.* 1278 341–352. 10.1007/978-1-4939-2425-7_21 25859960

[B52] Wu D. Cerutti C. Lopez-Ramirez M. A. Pryce G. King-Robson J. Simpson J. E. (2015). Brain endothelial miR-146a negatively modulates T-cell adhesion through repressing multiple targets to inhibit NF-kappaB activation. *J. Cereb. Blood Flow Metab.* 35 412–423. 10.1038/jcbfm.2014.207 25515214 PMC4348377

[B53] Yang C. Hawkins K. E. Dore S. Candelario-Jalil E. (2019). Neuroinflammatory mechanisms of blood-brain barrier damage in ischemic stroke. *Am. J. Physiol. Cell Physiol.* 316 C135–C153. 10.1152/ajpcell.00136.2018 30379577 PMC6397344

[B54] Yang Q. Tong X. Schieb L. Vaughan A. Gillespie C. Wiltz J. L. (2017). Vital Signs: recent Trends in Stroke Death Rates - United States, 2000–2015. *MMWR Morb. Mortal Wkly Rep.* 66 933–939. 10.15585/mmwr.mm6635e1 28880858 PMC5689041

[B55] Zhang C. (2008). The role of inflammatory cytokines in endothelial dysfunction. *Basic Res. Cardiol.* 103 398–406. 10.1007/s00395-008-0733-0 18600364 PMC2705866

[B56] Zhang L. Yang J. Xue Q. Yang D. Lu Y. Guang X. (2016). An rs13293512 polymorphism in the promoter of let-7 is associated with a reduced risk of ischemic stroke. *J. Thromb. Thrombolysis* 42 610–615. 10.1007/s11239-016-1400-1 27530126

[B57] Zhou J. Liu J. Pan Z. Du X. Li X. Ma B. (2015). The let-7g microRNA promotes follicular granulosa cell apoptosis by targeting transforming growth factor-beta type 1 receptor. *Mol. Cell Endocrinol.* 409 103–112. 10.1016/j.mce.2015.03.012 25817543

[B58] Zhuang Y. Peng H. Mastej V. Chen W. (2016). MicroRNA regulation of endothelial junction proteins and clinical consequence. *Media. Inflamm.* 2016:5078627. 10.1155/2016/5078627 27999452 PMC5143735

