# Supplementary material for: Let-7g* and miR-98 Reduce Stroke-Induced Production of Proinflammatory Cytokines in Mouse Brain
Source: Front Cell Dev Biol. 2020 Jul 17;8:632. doi: 10.3389/fcell.2020.00632 (PMC7379105; doi:10.3389/fcell.2020.00632)
Supplement: Supplementary file 1 [file Table_1.DOCX]

**
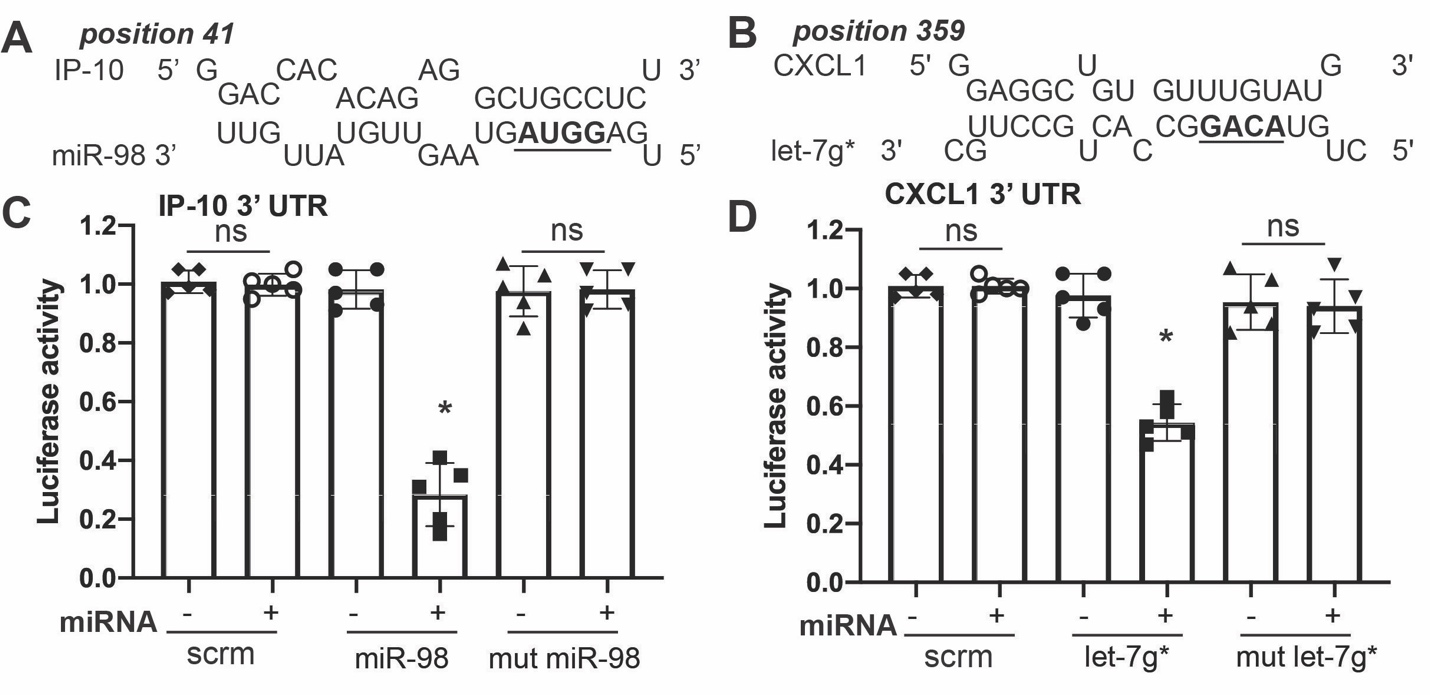
**

**Supplemental Figure 1.** *let-7g* and miR-98 selectively target CXCL1 and IP-10 3’ UTRs.* Prediction analysis for miR-98 and let-7g* for the ability to create miR-mRNA hybrid with IP-10 (A) and CXCL1 (B) 3’ UTRs, respectively. Luciferase activity for IP-10’s 3’ UTR reporter in HEK-293 cells transfected with wild-type and mutant forms of miR-98 (C) or let-7g* (D). Mutated nucleotides in the core sequence of miRs are bolded and underlined. Data are shown as mean ±SD. * = p < 0.05.
